# Errors in Text

**DOI:** 10.1001/jamanetworkopen.2020.29099

**Published:** 2020-11-20

In the Invited Commentary titled “Are Observational, Real-World Studies Suitable to Make Cancer Treatment Recommendations?”^1^ published July 30, 2020, there were errors in 6 parts of the text where the terms *propensity score–matched* and *propensity score–matching *were incorrectly used. The terms should have instead been *propensity-weighted *and* propensity score–weighting, *respectively. This article has been corrected.^1^
